# The mouse Gene Expression Database (GXD): 2014 update

**DOI:** 10.1093/nar/gkt954

**Published:** 2013-10-25

**Authors:** Constance M. Smith, Jacqueline H. Finger, Terry F. Hayamizu, Ingeborg J. McCright, Jingxia Xu, Joanne Berghout, Jeff Campbell, Lori E. Corbani, Kim L. Forthofer, Pete J. Frost, Dave Miers, David R. Shaw, Kevin R. Stone, Janan T. Eppig, James A. Kadin, Joel E. Richardson, Martin Ringwald

**Affiliations:** The Jackson Laboratory, 600 Main Street, Bar Harbor, ME 04609, USA

## Abstract

The Gene Expression Database (GXD; http://www.informatics.jax.org/expression.shtml) is an extensive and well-curated community resource of mouse developmental expression information. GXD collects different types of expression data from studies of wild-type and mutant mice, covering all developmental stages and including data from RNA *in situ* hybridization, immunohistochemistry, RT-PCR, northern blot and western blot experiments. The data are acquired from the scientific literature and from researchers, including groups doing large-scale expression studies. Integration with the other data in Mouse Genome Informatics (MGI) and interconnections with other databases places GXD’s gene expression information in the larger biological and biomedical context. Since the last report, the utility of GXD has been greatly enhanced by the addition of new data and by the implementation of more powerful and versatile search and display features. Web interface enhancements include the capability to search for expression data for genes associated with specific phenotypes and/or human diseases; new, more interactive data summaries; easy downloading of data; direct searches of expression images via associated metadata; and new displays that combine image data and their associated annotations. At present, GXD includes >1.4 million expression results and 250 000 images that are accessible to our search tools.

## INTRODUCTION

The laboratory mouse serves as a premier animal model in studying the complex molecular mechanisms that underlie the processes of human development, differentiation and disease. Tissues from all stages of mouse development and from many different mouse strains and mutants are being subjected to detailed expression analysis. The Gene Expression Database (GXD) collects these data from disparate sources, integrates them and makes them readily accessible to many types of biologically and biomedically relevant database searches.

By capturing multiple types of mRNA and protein expression information, including data from RNA *in situ* hybridization, immunohistochemistry, *in situ* reporter (knock in), reverse transcriptase-polymerase chain reaction (RT-PCR), northern blot and western blot experiments, GXD aims to provide increasingly complete information about where, when and in what amounts transcripts and proteins are expressed during development, as well as how their expression varies in different mouse strains and mutants. Data are acquired from the literature and from researchers, in particular from groups doing large-scale expression studies. All these data are annotated by GXD curators, making extensive use of controlled vocabularies and ontologies to provide the standardization of data that enables data integration and thereby complex queries. GXD forms an integral component of the larger Mouse Genome Informatics (MGI) resource. Through this association, the expression data can be combined with extensive genetic, functional, phenotypic and disease-orientated data (1). This robust integration, as well as interconnections with other resources (2–16), puts the expression data in GXD into a much larger analytical context.

Owing to its broad scope, thorough approach, data integration and querying capabilities, GXD provides an important and unique resource to the research community. GXD and its user interfaces have been described previously (17–20). Here we focus on recent progress in terms of data acquisition and improvements to the querying capabilities and web displays.

## DATA CONTENT AND PROGRESS IN DATA ACQUISITION

### Detailed expression data

**Figure 1.**
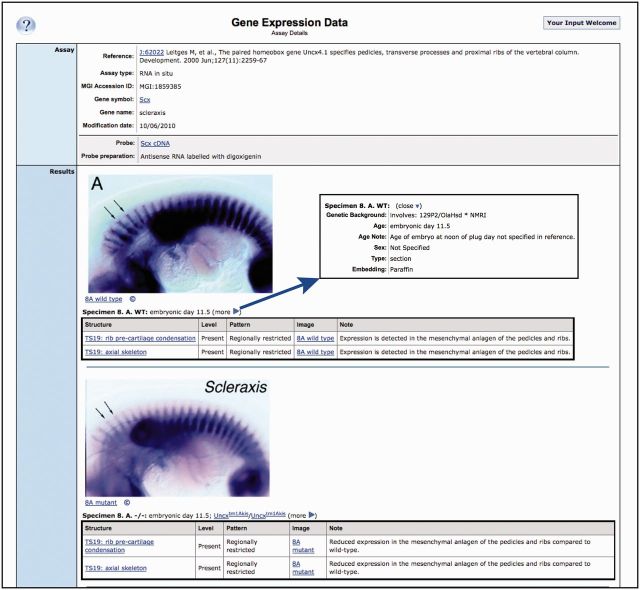
GXD assay details pages contain detailed expression annotations. This record for an RNA *in situ* hybridization assay illustrates the details included in GXD annotations of expression results. The Assay section reports the reference from which the data were derived, the assay type and the gene analyzed. Details regarding the nucleotide probe (or antibody) used in the assay can be accessed via the link on the page. In the ‘Results’ section, the Theiler stage and tissue examined, as well as the strength and pattern of expression as described by the author, are reported. (If this were a blot assay, the number and sizes of detected bands would also be reported.) Images of the original expression data are displayed beside the annotations describing them, allowing them to be reviewed in context. Major specimen details, such as the age and mutant alleles, are always displayed on the page. Other details, such as genetic background, sex and preparation method, can be viewed by using the ‘more’ toggle to expand that portion of the page. Assay details pages are accessed from the GXD data summaries.

GXD provides detailed records of expression results. The core entry is an assay details record (Figure 1). Each assay details record includes information about the gene studied, the probes and experimental conditions used, the specimen(s) analyzed, the expression results obtained for each specimen, as well as images of the data when available. These data are annotated using standard nomenclature and ontologies and serve as integration points within the GXD and MGI database. Expression patterns are described using an extensive, hierarchically structured anatomical ontology. As well as allowing for the integration of expression results from assays with differing spatial resolution, the hierarchical nature of the ontology allows expression searches by anatomical term to include all substructures for the term. The developmental portion of the anatomical ontology was begun by our collaborators from the eMouseAtlas project (21) and is being extended and refined jointly with GXD; the postnatal part was developed by the GXD project (22).

Since our last report, the amount of expression data in GXD has increased significantly both due to the GXD curators’ annotation of data from the literature and through the incorporation of data obtained from large-scale expression projects. In all cases, the curators review the data and standardize it. When necessary, they work with laboratories to resolve issues pertinent to nomenclature and data inconsistencies. The most recently acquired large data sets include RNA *in situ* studies of gene expression in the day 14.5 embryo [GenePaint; (23)]; RNA *in situ* and immunohistochemistry studies of gene expression in the genitourinary tract [GUDMAP; (24)]; and RNA *in situ* studies of gene expression in the embryonic and adult mouse nervous system [BGEM; (25)]. The integration of these data into GXD greatly expands the research community’s ability to query these data, increasing their utility.

As shown in Table 1, GXD currently contains detailed expression data for nearly 13 800 genes. There are 1.4 million expression result annotations; 82% are from RNA *in situ* hybridization studies and 10% from RT-PCR studies. These results include data from >1850 mouse mutants, as well as numerous strains of wild-type mice. In addition, the database contains >250 000 images of primary expression data.

**Table 1.** Data content in GXD as of 4 September 2013

15 051	Genes studied in expression references
13 767	Genes with expression assay results
64 174	Expression assays
1 401 522	Expression assay results
251 739	Expression images
1851	Mouse mutants with expression data

### Comprehensive literature survey

GXD maintains a comprehensive and up-to-date index of the embryonic gene expression literature that can be searched using the Gene Expression Literature query (http://www.informatics.jax.org/gxdlit). The curators survey journals to find all published articles that describe endogenous gene expression and knock-in reporter studies done in the embryonic mouse. They then review the entire publication, including any supplemental material, and record the genes and ages analyzed along with the expression assay types used. The annotation process includes determining the official nomenclature for each gene analyzed to ensure that the data is associated with the correct gene in the database. The assay types recorded are the same as those for which we have detailed expression annotations: RNA *in situ* hybridization, immunohistochemistry and knock-in (reporter) studies and blot assays. These annotations are combined with bibliographic information obtained from PubMed. The literature index allows researchers to identify publications that report specific sets of expression data, while helping the GXD staff prioritize publications for detailed expression curation. Currently the literature index covers >21 300 references analyzing >15 000 genes.

## KEY IMPROVEMENTS TO THE GXD USER INTERFACE

Users primarily access GXD via web-based search forms and displays. Searches return data summaries that in turn lead to the detailed expression assay records. We have redesigned the web pages at all three levels and reimplemented the underlying query mechanisms, thus generating a more powerful and versatile user interface.

### New search forms to access expression data

The Gene Expression Data Query (http://www.informatics.jax.org/gxd) provides access to the detailed expression data in GXD. It has been redesigned so that the two search utilities, ‘standard’ and ‘differential’, are accessed via tabs on the form. Both utilities are designed to be easy to understand and use.

**Figure 2.**
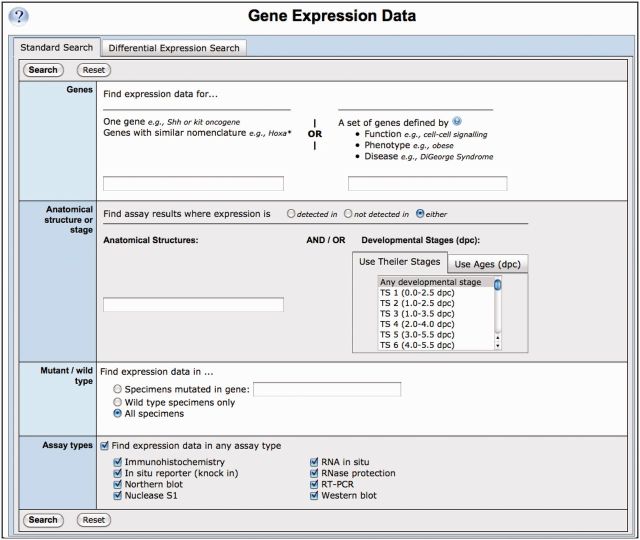
The Gene Expression Data Query form provides two search utilities via tabs. The Standard Search tab shown here allows users to query for expression data using one or more parameters. Searchable fields include gene symbol/name (and synonyms); anatomical structure; developmental stage/age; and assay type. The mutant/wild-type field can be used to limit searches to expression data obtained from mutants or to exclude data obtained from mutants. Integration with the other data in MGI enables searching of expression data by sets of genes that are defined by function [GO; (26)], phenotype [Mammalian Phenotype; (27)] or human disease [OMIM; (10)] terms. The Differential Expression Search tab allows biologists to search for genes that are expressed in some anatomical structures but not others and/or in some developmental stages but not others.

The Standard Search tab is shown in Figure 2. This form has been redesigned with new search functions. The Genes section now clearly delineates the options to search by gene: either based on gene nomenclature or by ontology terms that classify sets of genes. GXD users have long been able to use gene function [Gene Ontology (GO); (26)] terms for expression searches. We have added the capability to define sets of genes based on mammalian phenotype (27) or human disease association [Online Mendelian Inheritance in Man (OMIM); (10)]. Auto fill lists for these categories and for anatomical structure searches have been added to make it easy to find the appropriate search terms. Specimen age joins the previously available search by developmental (Theiler) stage as a search parameter.

The Differential Expression Search tab provides the functionality that was formerly found on the Gene Expression Data Expanded Query. It allows researchers to query for genes that are expressed in some anatomical structures but not others and/or in some developmental stages but not others. The redesigned search form has been streamlined to more clearly delineate its intended utility and different search capabilities.

### New data summaries

**Figure 3.**
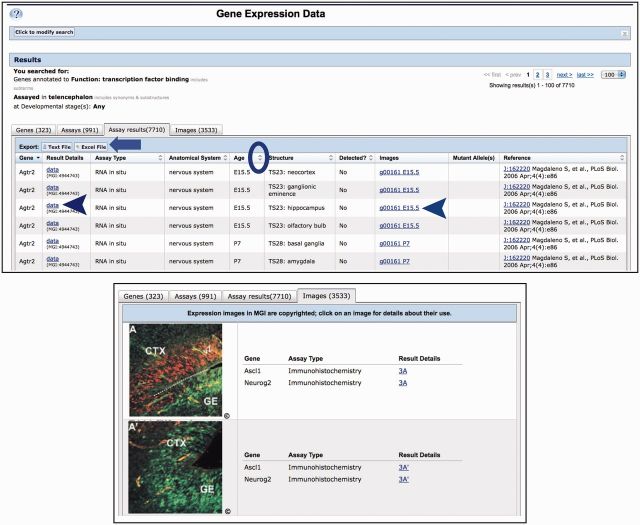
GXD’s data summaries are detailed, interactive and incorporate images. Each search for detailed expression data returns a page with tabbed summaries for the assay results, assays, genes and images that match the search parameters. The assay results tab (upper) lists the symbol of the gene examined, provides links (indicated by arrowheads) to the result details (such as those shown in Figure 1), lists the assay type used, lists the specimen age and tissue examined (reported as an anatomical system, age and anatomical structure), indicates whether expression was detected, provides a link to accompanying images, lists allele pairs describing the mutant genotype of the specimen (if applicable) and indicates the reference the data were derived from. The up and down arrows in the column header indicate that column is sortable; one set of arrows is circled. The assay results tab (as well as the genes tab) has buttons (indicated by the arrow) that allow for the export of results in text or spreadsheet format. The images tab (lower) allows users to quickly review images of the expression data that match their search criteria. The image is displayed beside a listing of the gene(s) examined in that image, the assay type used and a link to the result details. The ‘Click to modify search’ button near the top of the summary page (shown upper) reopens the search form, allowing for further refinement.

Each search for detailed expression data now returns a page with four tabbed summaries, one each for the assay results, assays, genes and images that match the search parameters (Figure 3). Thus, users can ‘zoom in and out’ to select the desired level of data granularity in the summary detail. The images tab, as well as the addition of the images column in the assay results tab, allows users to directly access and review images of the expression data that match their search criteria. Other new columns, such as anatomical system and genome location, have been added to the summaries to provide additional context to the returned expression results. Most columns on these summaries are sortable by clicking on arrows found in the headers. Export features have been added to the assay results and genes tabs to allow results to be downloaded in text or spreadsheet format. The genes list can be directly forwarded to the MGI batch query (http://www.informatics.jax.org/batch) to search for additional information, such as the function, phenotype and/or human disease terms associated with the genes of interest.

### New assay details pages

In GXD, data summaries link to the assay details expression records (Figure 1). The layout of these pages has been modified to emphasize the most important annotation details. The most salient specimen details such as the age and mutant alleles are displayed on the page, while other details, such as genetic background, sex and preparation method, can be viewed by using a toggle to expand that portion of the page. More importantly, however, the expression images have been integrated into the assay details pages, so they can be viewed together with their annotated results. Previously, the images were only displayed on image detail pages, which display the images as they are shown in their publications. Since each image is often part of a multipaned figure, this meant it could be difficult to compare the image with its expression annotations. Therefore, we segmented the multipaned figures into individual panes. This allowed each image pane to be displayed together with its annotations and also allowed these images to be made directly accessible to searches, as discussed above. However, because it is important to provide the scientific context for each image, the multipaned figures will continue to be available on the image detail page, which can be accessed by clicking on the images or image links on the assay details pages.

### New infrastructure for the web interface

In addition to redesigning the web interface, we have also dramatically improved its performance. The database tables used to generate the web displays have been de-normalized and optimized for these displays. Solr/Lucene indexes are now generated to provide fast and powerful text searching, as well as fast paging through large sets of results. The web interface itself has been reimplemented in Java using the Spring model-view-controller (MVC) framework running in JBoss; the Yahoo user interface library (YUI) is used as a Javascript framework. Hibernate is used for the data model and database access. Owing to these software infrastructure improvements, it is now possible to return all matching results for any search quickly; even all the database’s expression results can be returned in a short period.

### GXD BioMart

As another option to access GXD data, we have installed a GXD BioMart application (http://biomart.informatics.jax.org). BioMart is a data management and integration system that performs like a batch query (28). It also supports iterative queries. Based on the filters selected, users can return expression data for a gene or a list of genes, an anatomical structure, a Theiler stage, a mutant and/or a reference. The search results can be further customized based on attributes chosen by users, and then they can be saved as HTML or downloaded as comma- or tab-separated files. Importantly, the GXD BioMart can also be interconnected with other BioMarts, enabling queries across the combined resources.

## USER SUPPORT

GXD provides support to its users through a dedicated User Support staff, detailed online documentation and FAQs. User Support can be contacted via email at mgi-help@jax.org or by clicking the User Support link at the bottom of our web pages. The online documentation can be accessed by clicking on the question mark in the upper left corner of most pages. FAQs (and other useful links) can be found on the GXD home page (http://www.informatics.jax.org/expression.shtml).

## CITING GXD

The following citation format is suggested when referring to data downloaded from GXD: These data were retrieved from the GXD, MGI, The Jackson Laboratory, Bar Harbor, Maine, USA (URL: http://www.informatics.jax.org) on [type in date (month, year) when you retrieved the data cited]. To reference the database itself, please cite this article.

## FUNDING

Eunice Kennedy Shriver National Institute of Child Health and Human Development (NICHD) of the National Institutes of Health (NIH) [HD062499]; The implementation of the GXD BioMart was supported by the European Commission [project number 223592]. Funding for open access charge: NIH [HD062499].

*Conflict of interest statement*. None declared.
